# Skin organoid-derived exosomes in a hydrogel system potentiate skin wound healing

**DOI:** 10.1016/j.mtbio.2026.103632

**Published:** 2026-09-01

**Authors:** Tao Yang, Wen Zheng, Yue Li, Yingshi Li, Zhuxian Wang, Qiuyu Chen, Yangmeihui Wang, Xiaoye Shi, Jun Liu, Bin Yang

**Affiliations:** a Dermatology Hospital, Southern Medical University, Guangzhou, People's Republic of China; b The First School of Clinical Medicine, Southern Medical University, Guangzhou, People's Republic of China; c Department of Dermatology, The First Affiliated Hospital of Anhui Medical University, Hefei, People's Republic of China

**Keywords:** Skin organoids, Exosome, Hydrogel, Wound healing, Neovascularization

## Abstract

Skin wounds, whether acute or chronic, remain a major clinical challenge due to their complex healing processes involving inflammation, angiogenesis, and tissue remodeling. While stem cell-derived exosomes offer a promising cell-free therapeutic approach, their clinical translation is limited by rapid clearance and insufficient tissue-specificity. In this study, we developed a novel strategy by integrating exosomes derived from skin organoids (SKO-Exo)—which mimic native skin heterogeneity—into a hydrogel-based sustained-release system (Exo-Gel) to enhance wound healing. Skin organoids were generated from human embryonic stem cells, and exosomes were isolated and characterized, revealing typical cup-shaped morphology and exosomal markers. The hydrogel, composed of polyvinyl alcohol/gelatin/boric acid, exhibited excellent self-healing, adhesion, and sustained exosome release with an initial burst. In vitro, SKO-Exo-loaded hydrogel (SKOexo-Gel) significantly promoted endothelial cell proliferation, migration, and tube formation, as well as fibroblast collagen III secretion, outperforming exosomes from 3D keratinocytes (KC-Exo). In a rat full-thickness wound model, SKOexo-Gel accelerated wound closure, enhanced angiogenesis, and increased collagen III deposition. miRNA sequencing identified Hsa-miR-125b-5p as a prominent SKO-Exo-enriched miRNA that activates the Wnt/β-catenin pathway to drive angiogenesis, validated by functional assays. These findings demonstrate that skin organoid-derived exosomes in a hydrogel system synergistically promote wound healing through tissue-specific cues, offering a next-generation therapy for refractory wounds.

## Graphical abstract

**Figure:**
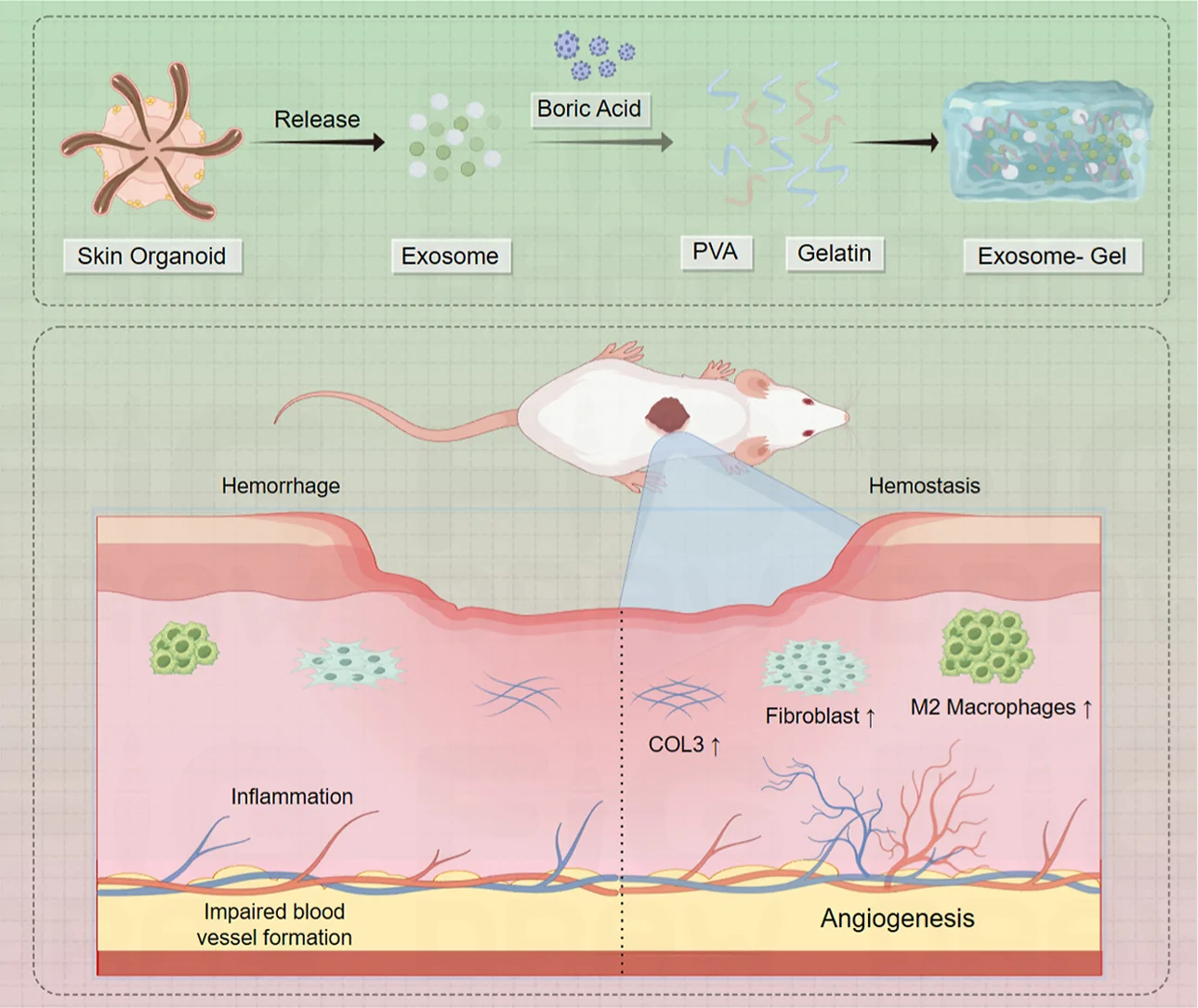

## 1 Introduction

Skin wounds, including both acute injuries (such as burns and traumatic defects) and chronic conditions (such as diabetic ulcers), pose significant challenges in clinical practice due to their complex healing processes involving inflammation, cell proliferation, angiogenesis, and tissue remodeling [1]. Conventional treatments often fail to achieve functional recovery, leading to high socioeconomic burdens and impaired patient quality of life [2]. In recent years, exosomes derived from stem cells have emerged as a promising cell-free therapy for wound repair [3]. These nano-sized extracellular vesicles (30–150 nm) carry bioactive molecules (e.g. proteins, miRNAs, and lipids) that regulate intercellular communication, promote angiogenesis, accelerate epithelialization, and modulate immune responses [4]. Compared to direct stem cell therapy, exosomes offer advantages including reduced immunogenicity, avoidance of ethical concerns, and superior stability during storage and transportation [5,6].

However, the clinical translation of exosome-based therapies is limited by rapid clearance in vivo, short half-life, and insufficient local retention at the wound site [7]. To address these issues, hydrogel-based sustained-release systems have been developed as effective carriers for exosomes. Hydrogels, such as gelatin methacryloyl (GelMA) or extracellular matrix (ECM)-derived hydrogels, provide a biocompatible and degradable scaffold that protects exosomes from degradation and enables controlled release, thereby prolonging their therapeutic activity [8,9]. For instance, studies combining mesenchymal stem cell (MSC)-derived exosomes with hydrogels have demonstrated enhanced wound closure, collagen deposition, and vascularization in animal models [10,11]. Beyond these foundational applications, sophisticated multifunctional systems have been engineered to tackle complex pathological conditions. Notably, Xiong et al. developed an HA-based hydrogel co-delivering M2 macrophage-derived exosomes and FGF-2 with MnO_2_ nanozymes to concurrently address bacterial infection, oxidative stress, and angiogenesis deficiency in diabetic ulcers, serving as a benchmark for multifunctional exosome-hydrogel design [12].

Despite these advances, the biological source of exosomes critically influences their therapeutic efficacy, yet the core limitation of current exosome therapies lies in the suboptimal alignment between exosomal cargo and the multifaceted demands of skin repair [13]. Most current studies rely on exosomes from MSCs or bodily fluids, whose cargos are evolutionarily programmed to support the homeostasis of their origin tissues (e.g., MSC exosomes prioritize skeletal/cartilage regeneration or broad immunosuppression) rather than the multi-stage, cell-type-specific programs required for skin repair. Even exosomes from isolated skin cell types (e.g., keratinocytes or fibroblasts) fail to recapitulate the coordinated intercellular signaling that governs native skin regeneration: for example, keratinocyte-derived exosomes (KC-Exo) predominantly carry epithelial-specific signals that promote re-epithelialization but lack cues for dermal matrix remodeling [14]. Recently, organoid-derived exosomes have gained attention for their ability to mimic the multicellular composition and functional characteristics of original organs [15]. For example, salivary gland organoid-derived exosomes exhibit protein profiles similar to human saliva and accelerate healing by inducing macrophage polarization and angiogenesis [16]. Critically, skin organoids recapitulate not only the cellular heterogeneity of native skin (including keratinocytes, fibroblasts, hair follicle lineages, and sebaceous glands) but also the developmental signaling cascades (e.g., Wnt/β-catenin-mediated epithelial-mesenchymal crosstalk) that govern skin morphogenesis processes absent in single-cell cultures or non-skin-derived stem cells [[17], [18], [19]]. This unique developmental programming suggests skin organoid-derived exosomes may carry a “tissue-native regenerative code” that simultaneously coordinates epithelialization, angiogenesis, and scarless matrix remodeling, a capability unmatched by existing exosome sources. These exosomes potentially offer superior efficacy in coordinating multiple phases of wound healing compared to those from homogeneous cell cultures.

Nevertheless, studies on exosomes from pluripotent stem cell (PSC)-derived multilineage skin organoids remain scarce. Prior applications of skin organoids in wound healing have primarily focused on intact organoid transplantation: landmark work demonstrated that transplanted skin organoids accelerate full-thickness wound closure and reduce scarring [19]. However, whole-organoid grafting faces well-documented translational barriers: surgical invasiveness, potential immunogenicity, batch-to-batch heterogeneity, and challenges in scalable manufacturing. As a cell-free alternative, skin organoid-derived exosomes are expected to circumvent these limitations while retaining the tissue-specific regenerative cues of native skin. Notably, no prior study has: (1) systematically compared SKO-Exo with exosomes from homogeneous skin cell cultures to validate the advantage of multicellular heterogeneity; (2) integrated SKO-Exo with a sustained-release hydrogel system to address the challenges of in vivo therapeutic delivery; or (3) dissected the molecular cargos and functional mechanisms underlying SKO-Exo-mediated repair. Our work fills these critical gaps.

In this study, we developed a novel wound healing strategy by integrating exosomes derived from skin organoids into a hydrogel-based sustained-release system. Our approach aims to leverage the dual advantages of skin organoid exosomes, which carry tissue-specific regenerative signals and the hydrogel ability to provide localized, controlled exosome delivery. Using a rat full-thickness wound model, we demonstrated that this composite system significantly promotes wound closure, collagen synthesis, and angiogenesis, while the hydrogel matrix itself facilitates macrophage polarization toward the M2 phenotype. Our findings highlight the potential of skin organoid exosome hydrogel hybrids as a next-generation therapy for skin wounds.

## 2 Materials and methods

### 2.1 Generation of skin organoids and 3D keratinocytes

Skin organoids were differentiated from human embryonic stem cells (hPSCs) following an established protocol with modifications [20]. Briefly, hESCs were aggregated and sequentially induced under defined conditions: initially treated with a TGF-β inhibitor, BMP4, and a low dose of bFGF to specify surface ectoderm, followed by BMP inhibition and a high dose of bFGF to promote cranial neural crest cell formation and subsequent skin morphogenesis. The cultures were maintained in a rotating bioreactor for up to 130 days, during which time the organoids developed stratified epidermis, dermal layers, and appendages such as hair follicles. Morphological changes and the emergence of key structures like hair germs were routinely monitored by bright-field microscopy. For the 3D keratinocyte model, human keratinocytes were embedded in Matrigel to form stratified epithelial spheres.

### 2.2 Exosome isolation and characterization

Exosomes were isolated from the conditioned media of skin organoids (SKO-Exo) and 3D keratinocytes (KC-Exo) via differential ultracentrifugation, a widely accepted gold-standard method for exosome isolation [21]. Briefly, the conditioned media were subjected to sequential centrifugation steps at 300 × g for 10 min, 2000 × g for 20 min, and 10,000 × g for 30 min to remove cells, debris, and large vesicles. The supernatant was then ultracentrifuged at 100,000 × g for 70 min (Beckman Coulter Optima XPN-100, SW 32 Ti rotor) to pellet exosomes. The crude exosome pellet was washed once in PBS and subjected to a second ultracentrifugation at 100,000 × g for 70 min to improve purity. The final exosome pellets were resuspended in PBS and stored at −80°C until further use.

Characterization involved transmission electron microscopy (TEM) for morphology, nanoparticle tracking analysis (NTA) for size distribution, and western blotting for both positive exosomal markers (TSG101, CD63, Alix) and negative markers (Calnexin, GM130) to confirm exosome identity and exclude cellular contamination. For uptake assays, labeled exosomes were incubated with HUVECs, HDFs, and HaCaTs, and internalization was visualized by fluorescence microscopy.

### 2.3 Fabrication and characterization of the exosome-loaded hydrogel

To ensure fair comparison between KC-Exo and SKO-Exo in downstream experiments, exosomes were quantified based on particle number using NTA. The particle concentration (particles/mL) was measured for each preparation, and equal particle numbers were used for subsequent in vitro and in vivo assays. For hydrogel loading, a defined quantity of 2 × 10^11^ particles was incorporated per formulation. Protein content was also measured by BCA assay for reference, but particle number was used as the primary normalization method.

The exosome-loaded hydrogel was fabricated using a polyvinyl alcohol (PVA)/gelatin/boric acid system [22]. Briefly, 8 g of PVA was swollen in 65 g of water for at least 4 h, and 2 g of gelatin was separately swollen in 35 g of water for 15 min. Both solutions were then completely dissolved by stirring at 85°C. The dissolved PVA and gelatin solutions were cooled to 30-40°C and mixed thoroughly. Separately, 0.5 g of boric acid was dissolved in 50 g of water at 85°C and cooled to 30-40°C. A defined quantity of exosomes (2 × 10^11^ particles) was mixed into the boric acid solution. Finally, 16 g of the PVA/gelatin mixture was rapidly combined with 4.5 g of the exosome-containing boric acid solution under stirring to induce crosslinking and gel formation. The resulting Exo-Gel was stored at 4°C for subsequent use. The hydrogel's porous structure was examined by scanning electron microscopy (SEM). Rheological properties (storage modulus G′ and loss modulus G″) were measured via amplitude and frequency sweeps. Self-healing was assessed by rejoining bisected hydrogel pieces, and adhesion was tested on fresh biological tissues.

The release profile of exosomes from the hydrogel was evaluated under physiological conditions (37°C, PBS, pH 7.4). To accurately capture the initial burst release, samples were collected every 6 h during the first 24 h, followed by daily sampling thereafter. A Blank-Gel (hydrogel without exosomes) was included as a control, and its absorbance at each time point was subtracted to correct for background interference from gelatin degradation products. Collected supernatants were centrifuged at 10,000 × g for 10 min to remove hydrogel debris, then incubated with 1% Triton X-100 to lyse exosome membranes prior to protein quantification using a BCA assay.

The antibacterial activity of the hydrogels was evaluated against *Staphylococcus aureus* (*S. aureus*), *Pseudomonas aeruginosa* (*P. aeruginosa*), and methicillin-resistant *Staphylococcus aureus* (MRSA). Single colonies were inoculated into Mueller-Hinton broth (MHB) and incubated at 37°C with shaking at 200 rpm for 12 h. Bacterial suspensions were adjusted to 10^6^ colony-forming units per mL (CFU/mL) based on optical density at 600 nm. Five experimental groups were then set up with group containing 10 μL of 10^6^ CFU mL/mL of bacterial suspension, prepared as above, together with 1) 990 μL PBS, 2) 990 μL of 4 μg/mL ampicillin (AM), 3)Blank-Gel coated 48-well plate, 4) KCexo-Gel coated 48-well plate, and 5) SKOexo-Gel coated 48-well plate. Each well were then incubated at 37°C for 2 h. After incubation, the bacteria were resuspended in 1 mL of PBS and plated on LB agar for 18 h at 37°C. Viable bacteria were quantified as CFU/mL.

### 2.4 In vitro cellular assays

2 × 10^3^ HUVECs, HDFs, and HaCaTs were pre-seeded into 96-well plate. After 24 h of incubation, the medium was refreshed, and the hydrogels were added. The proliferation were assessed using an EdU incorporation kit. Cell migration was evaluated using a scratch-wound assay, and images were taken at 0, 24, and 48 h to quantify closure. Tube formation assays were performed by seeding HUVECs on Matrigel and measuring total tube length and branch points. Collagen I and III secretion by HDFs was analyzed by immunofluorescence staining and quantitative intensity measurement.

### 2.5 Animal wound healing model

All animal experiments were conducted in accordance with the National Institutes of Health guidelines for the Care and Use of Laboratory Animals. Guangdong Huawi Testing Co., Ltd. Institutional Animal Care and Use Committee (IACUC) examined and approved animal experiments (Approval No. 202501006).

Adult male Sprague-Dawley (SD) rats, weighing 250–300 g, were randomly assigned to four groups (n = 6 per group): a Tegaderm film control group, a blank hydrogel group, a hydrogel loaded with 3D keratinocyte-derived exosomes group, and a hydrogel loaded with skin organoid-derived exosomes group. Following anesthesia via an intraperitoneal injection of pentobarbital (40 mg/kg), the dorsal hair was shaved, and the skin was disinfected. Full-thickness excisional wounds were created on the dorsum of each rat using a sterile disposable biopsy punch with a diameter of 1.5 cm. In the Blank-Gel, KCexo-Gel, and SKOexo-Gel groups, the corresponding hydrogel was applied evenly to cover the entire wound bed. The Tegaderm film group was covered with a sterile Tegaderm transparent film. The hydrogels and Tegaderm were refreshed every two days to ensure sustained exosome availability at the wound site. After surgery, all rats were individually housed in a controlled environment with free access to food and water. The wound healing process was monitored, and wounds were photographed on days 0, 2, 4, 6, 10, and 14 for subsequent analysis.

For the diabetic wound model, diabetes was first induced in adult male SD rats (250–300 g) by intraperitoneal injection of streptozotocin (STZ, 65 mg/kg) for five consecutive days. After 72 h of normal feeding, rats with fasting blood glucose levels exceeding 16.7 mmol/L were considered diabetic and used for subsequent experiments. Full-thickness excisional wounds (1.5 cm diameter) were created on the dorsum, the grouping and administration protocols were identical to those described above.

### 2.6 Hemostatic ability evaluation

In vitro whole blood coagulation assays were performed by incubating blood with hydrogels and measuring clotting time. For in vivo models, a liver incision and a tail amputation model were used. The hydrogel was applied to the wound, and blood loss was determined by weighing the gauze before and after application.

### 2.7 Histological and immunofluorescence analysis

Wound tissues harvested on day 7 and 14 were fixed in 4% paraformaldehyde for 24 h, paraffin-embedded, and sectioned at 5 μm thickness. For H&E staining, sections were deparaffinized in xylene (3 × 3 min), rehydrated through graded ethanol (100%, 95%, 70%, each 3 min), stained with Mayer's hematoxylin for 5 min, differentiated in 1% acid alcohol, and counterstained with 0.5% eosin Y for 1 min before dehydration and mounting. For collagen evaluation, Masson's trichrome staining was performed: after deparaffinization, sections were stained in Weigert's iron hematoxylin (10 min), immersed in acid fuchsin/ponceau solution (10 min), treated with 1% phosphomolybdic acid (5 min), and differentiated in 2% light green SF (10 min).

For immunofluorescence, antigen retrieval was performed using citrate buffer (pH 6.0, 95°C, 20 min). Sections were blocked with 5% BSA for 30 min and incubated overnight at 4°C with primary antibodies diluted in PBS as follows: anti-CD31 (1:200), anti-α-SMA (1:300), anti-iNOS (1:250), anti-CD206 (1:300), anti-Collagen I (1:400), and anti-Collagen III (1:400). After washing, sections were incubated with species-matched Alexa Fluor-conjugated secondary antibodies (e.g., goat anti-rabbit IgG Alexa Fluor 488/555, 1:500) for 1 h at room temperature, followed by DAPI (1 μg/mL, 5 min) for nuclear counterstaining. Images were acquired using a confocal microscope with consistent settings, and fluorescence intensity or positive cells were quantified using ImageJ software.

### 2.8 miRNA sequencing and bioinformatic analysis

Total RNA was extracted from SKO-Exo and KC-Exo. After quality assessment, qualified RNA samples were used for small RNA library construction and sequenced on an Illumina platform. Differential expression analysis was performed, and a volcano plot was generated. KEGG pathway enrichment analysis was conducted on the target genes of upregulated miRNAs in SKO-Exo.

Candidate miRNAs were validated by qPCR using three independent biological replicates of SKO-Exo and KC-Exo. Before RNA extraction, a fixed amount of cel-miR-39-3p was spiked into each exosome lysate as an exogenous normalization control. Total exosomal small RNA was quantified using the Qubit microRNA Assay, and equal masses were used for cDNA synthesis. Relative expression was calculated by the 2^(−ΔCt) method, where ΔCt = Ct(target) − Ct(cel-miR-39). A cutoff of qPCR-derived fold change (SKO-Exo/KC-Exo) ≥ 100-fold was applied to select candidate miRNAs for functional assays.

### 2.9 Functional validation of miRNAs

For functional validation, HUVECs at 60-70% confluency were transfected with 50 nM miRNA mimics or negative control using Lipofectamine 3000 (2.5 μL reagent/μg RNA). After 6 h incubation, the medium was replaced with fresh complete medium, and functional assays were conducted 48 h post-transfection. Cell proliferation was assessed using an EdU incorporation kit where transfected HUVECs were incubated with 10 μM EdU for 2 h, followed by fixation, permeabilization, and Click-iT reaction for quantification of EdU-positive cells. Migration was evaluated by scratch-wound assay using a sterile pipette tip to create linear scratches, with cells maintained in serum-reduced medium (2% FBS) and wound closure quantified at 0, 24, and 48 h using image analysis. Tube formation ability was assessed by seeding transfected HUVECs (2 × 10^4^ cells/well) on polymerized Matrigel (50 μL/well) and quantifying capillary-like structures after 6-8 h incubation. For mechanistic investigation, immunofluorescence staining for β-catenin was performed 48 h post-transfection involving fixation with 4% PFA, permeabilization with 0.1% Triton X-100, blocking with 3% BSA, and incubation with primary antibody (1:200) and secondary antibody (1:500) followed by DAPI counterstaining. Rescue experiments involved co-treatment of miR-125b-5p-transfected HUVECs with 5 μM MSAB (β-catenin inhibitor), with subsequent re-evaluation of proliferation and migration to determine pathway dependency.

### 2.10 Statistical analysis

Data are presented as mean ± SD. Statistical significance was determined using Student's t-test for two groups or one-way/two-way ANOVA with Tukey's post-hoc test for multiple comparisons. A p-value < 0.05 was considered significant. All analyses were performed using GraphPad Prism software.

## 3 Result

### 3.1 Characterization of exosomes derived from 3D keratinocytes and skin organoids

**Fig. 1:**
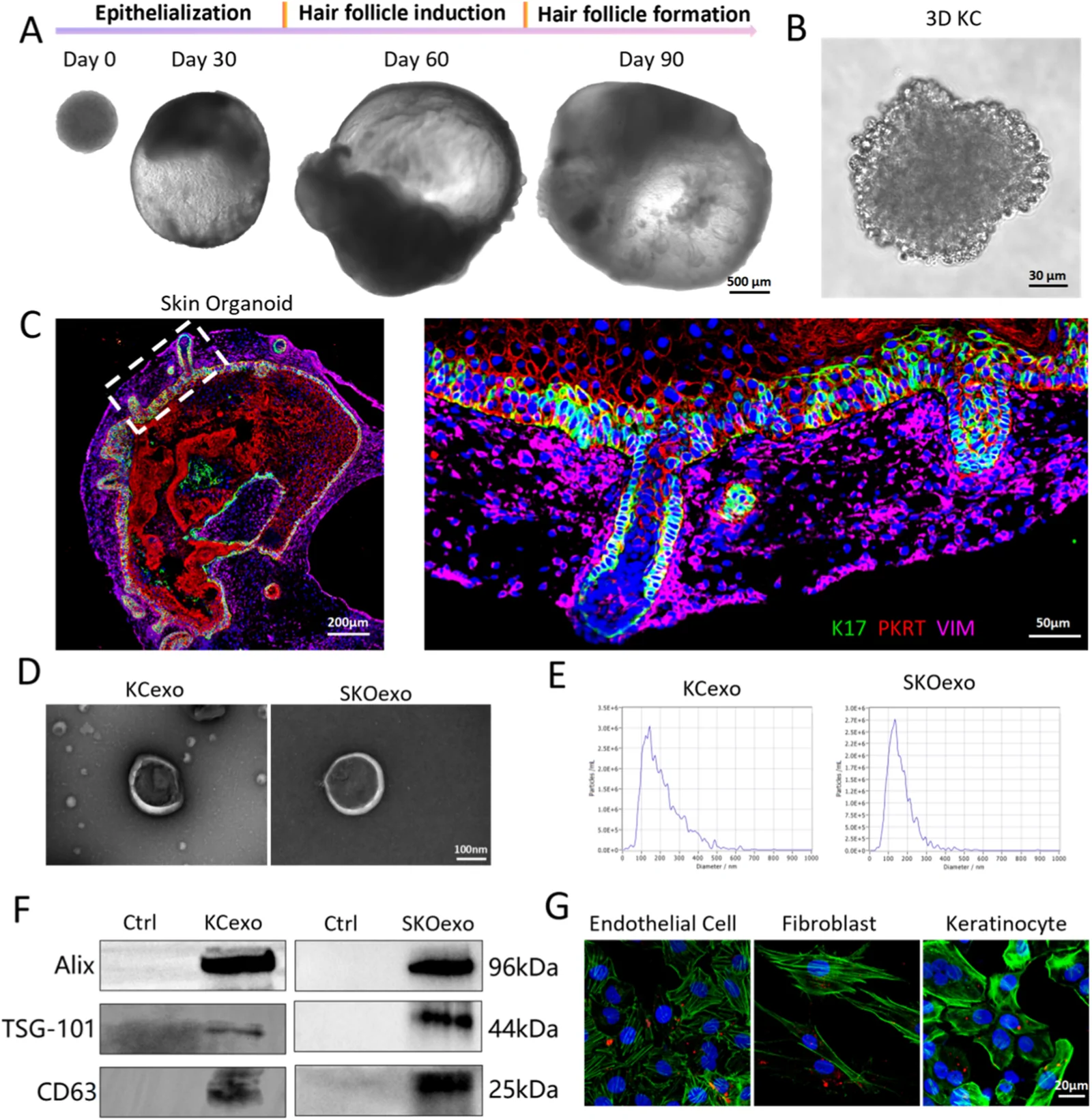
| **Characterization of skin organoid, 3D-derived keratinocytes and their exosomes. A**, Representative bright-field images illustrating the morphological progression of skin organoid cultures through sequential developmental stages: epithelialization (Days 0-30), hair follicle induction (Days 60), and mature hair follicle formation (Days 90). Scale bars are shown on the images. **B**, Representative bright-field images of 3D Keratinocytes showing their typical spheroidal structure. **C**, Confocal microscopy images of skin organoid: (left) a cross-sectional overview and (right) a magnified view of cellular assemblies, immunostained for keratin 17 (K17, green), pan-cytokeratin (PKRT, red), and vimentin (VIM, blue). Scale bars: 200 μm (left) and 50 μm (right).**D**, Transmission electron microscopy images of exosomes derived from 3D-derived keratinocytes and skin organoids, confirming their spherical, membrane-bound nanovesicle structure. Scale bar: 100 nm. **E**, Particle size distribution profiles of KCexo and SKOexo, as determined by dynamic light scattering. **F**, Western blot analysis validating the enrichment of canonical exosomal marker proteins (Alix, TSG-101, CD63) in both KCexo and SKOexo samples compared to the cell lysate control. **G**, Cellular uptake of fluorescently labeled exosomes (red) by primary human dermal microvascular endothelial cells, human dermal fibroblasts, and human keratinocytes (KCs) after co-incubation. F-actin (phalloidin, green) and nuclei (blue) are stained. Red punctate signals indicate the successful internalization of SKO-Exo into the three primary human skin cell types. Scale bar: 20 μm. (For interpretation of the references to color in this figure legend, the reader is referred to the Web version of this article.)

We successfully generated skin organoids from embryonic stem cells and established a 3D keratinocyte culture model. White-light microscopy of the developing organoids revealed a characteristic temporal progression. Initial embryoid bodies (EBs) underwent significant epithelialization by day 30. Subsequent hair follicle induction commenced around day 60, marked by the initial emergence of dermal papilla-like structures. By approximately day 90, the skin organoids reached maturation, exhibiting well-defined, mature hair follicle structures. Histological examination revealed that the skin organoids recapitulated the key structural features of native skin, including distinct epidermal and dermal layers, as well as developing skin appendages such as hair follicles. In contrast, the 3D keratinocyte model exhibited a comparatively simple structure, primarily consisting of stratified keratinocytes without the complexity of associated dermal components or appendages (Fig. 1A,B,C). Subsequently, exosomes were isolated from the conditioned media of both skin organoids and the 3D keratinocyte cultures, and characterized using standard methodologies. Transmission electron microscopy (TEM) imaging demonstrated that exosomes from both sources displayed the characteristic cup-shaped morphology, with diameters approximately ranging around 100 nm (Fig. 1D). Nanoparticle Tracking Analysis (NTA) further confirmed the size distribution of the isolated vesicles, showing a predominant peak within the typical exosomal size range (30-150 nm) for both preparations, indicating a homogeneous particle population (Fig. 1E). Furthermore, Western blot analysis was performed to assess the purity of the isolated exosomes. Established exosomal marker proteins, including the cytosolic protein TSG101, the transmembrane protein CD63, and Alix, were positively expressed in both KC-Exo and SKO-Exo samples (Fig. 1F). To further exclude cellular contamination, we examined the negative markers Calnexin (endoplasmic reticulum) and GM130 (Golgi apparatus). Calnexin and GM130 were detected in the cell lysate controls but were absent in the exosome samples, confirming the absence of significant organelle or cellular debris contamination and verifying the integrity of the isolated exosomes (Fig. S1). The exosome uptake experiment showed that endothelial cell, fibroblast and keratinocyte could effectively internalize the exosomes, as evidenced by fluorescence labeling (Fig. 1G).

### 3.2 Fabrication and characterization of the hydrogel-based wound healing system

**Fig. 2:**
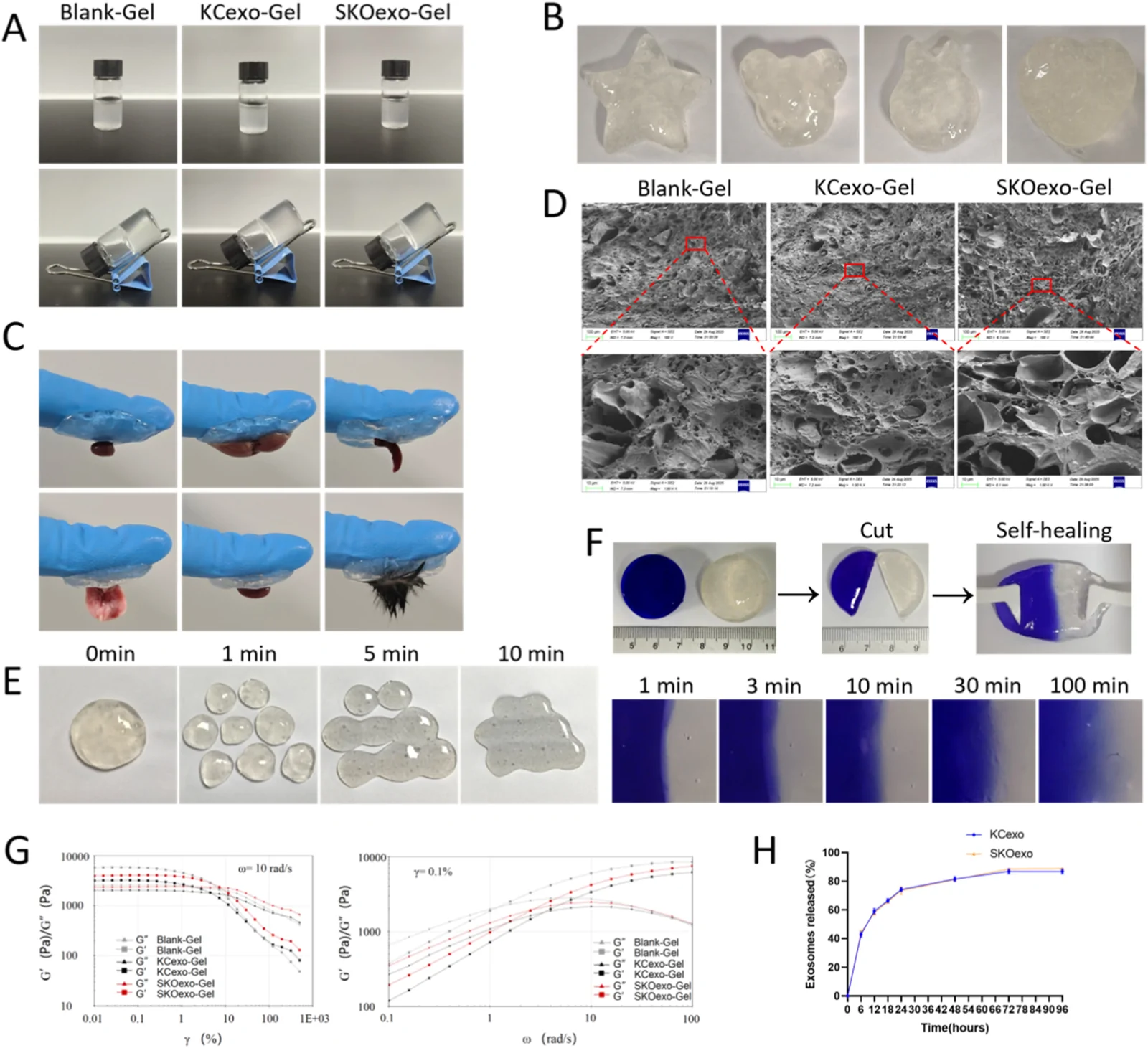
| **Comprehensive characterization of the exosome-embedded hydrogel. A**, Photographs illustrating the formation and transparency of Blank-Gel, KCexo-Gel, and SKOexo-Gel. **B**, Shape-remodeling capability and adaptability, showing the gels can be molded and retain specific structures. **C**, Qualitative adhesive capacity of the gels on various tissues. **D**, Representative scanning electron microscopy (SEM) images of the internal microstructures of the three gels. Red boxes indicate the magnified regions. Scale bars are shown on the images. **E,F** Macroscopic self-healing property observed over time. **G,** Dynamic oscillatory rheometry showing the storage modulus (G′) and loss modulus (G″) of the gels over successive cycles of cutting and reassembly, demonstrating mechanical resilience. **H,** Cumulative release profile of exosomes from KCexo-Gel and SKOexo-Gel over 4 days, measured in PBS at 37°C. Data are represented as mean ± SD (n = 3–4/group). *P < 0.05, **P < 0.01, ***P < 0.001. (For interpretation of the references to color in this figure legend, the reader is referred to the Web version of this article.)

We developed a versatile hydrogel system for the sustained release of exosomes (Exo-Gel). Initial characterization confirmed its key physicochemical and biological properties. The prepared hydrogels exhibit translucent appearance and a consistent gel-like state. To assess the short-term structural stability, samples were tilted at an angle. Over a 5-min observation period, the hydrogels maintained their shape without significant flow or deformation, demonstrating sufficiently high viscosity and stability against gravitational stress. Notably, no apparent differences in color, translucency, or flow behavior were observed between the blank hydrogel and the Exo-Gel, indicating that the incorporation of exosomes did not alter these fundamental physical properties of the hydrogel matrix (Fig. 2A). To evaluate the material's ability to conform to complex contours, the hydrogel was manually remodeled into a series of distinct shapes. The successful reformation into these varied geometries without structural failure demonstrates the excellent plasticity and shape adaptability of the Gel. This property indicates its potential to effectively fill and conform to irregular wound beds, a crucial characteristic for clinical application in wound management (Fig. 2B). The Gel demonstrated robust adhesion, forming a conformal interface with the surfaces of various freshly excised biological tissues. The observed robust adhesive performance establishes the Gel as a reliable physical sealant, suggesting its potential utility for hemostasis (Fig. 2C). Scanning electron microscopy (SEM) revealed a uniformly porous microstructure (Fig. 2D), which is conducive to nutrient exchange and cell infiltration. This well-defined, interconnected porous microstructure is a critical feature that is expected to facilitate cell migration, vascularization, and effective integration with surrounding tissue in wound healing applications. The hydrogel demonstrated rapid self-healing, as segments of a bisected sample autonomously re-joined upon contact. Dye diffusion assays revealed substantial molecular mobility across the damaged interface, with significant migration observable within 3 min and near equilibrium by 100 min. This rapid interdiffusion indicates efficient polymer chain re-entanglement at the healing junction, underpinning the macroscopic self-healing behavior (Fig. 2E and F).

The viscoelastic behavior of Blank-Gel, KCexo-Gel, and SKOexo-Gel was characterized via oscillatory shear measurements. Strain amplitude sweeps at a fixed frequency (ω = 10 rad/s) revealed that all formulations transitioned from a solid-like dominant region (G' > G″) to a crossover point and eventual liquid-like behavior (G" > G′) at similar strain amplitudes. Frequency sweeps at a fixed strain (γ = 0.1%) within the linear viscoelastic region showed that all hydrogels exhibited qualitatively similar viscoelastic solid behavior, with G′ consistently greater than G″ across the measured frequency range. Critically, the G′ and G″ profiles for KCexo-Gel and SKOexo-Gel closely overlapped with those of the Blank-Gel in both tests. This indicates that the incorporation of exosomes did not significantly alter the fundamental linear viscoelastic properties or the network relaxation dynamics of the base hydrogel matrix (Fig. 2G). Critically, the Exo-Gel demonstrated an initial burst release within 24 h, followed by a sustained release profile that stabilized at approximately 85% over 96 h, consistent with the physical entrapment-dominated retention mechanism reported for analogous PVA/gelatin/borax hydrogel systems. (Fig. 2H). Notably, in vitro antibacterial assays demonstrated that the Blank-Gel and exosome-loaded hydrogels neither inhibited nor promoted the growth of *S. aureus*, *P. aeruginosa*, and MRSA compared to the PBS control, highlighting the necessity of regular hydrogel replacement during in vivo treatment to prevent bacterial accumulation at the wound site (Fig. S2). Collectively, these results demonstrate that the developed Gel possesses desirable porous morphology, self-healing, and sustained exosome release properties, making it a promising candidate for wound healing applications.

### 3.3 In vitro evaluation of cellular responses promoted by the exosome-loaded hydrogels

**Fig. 3:**
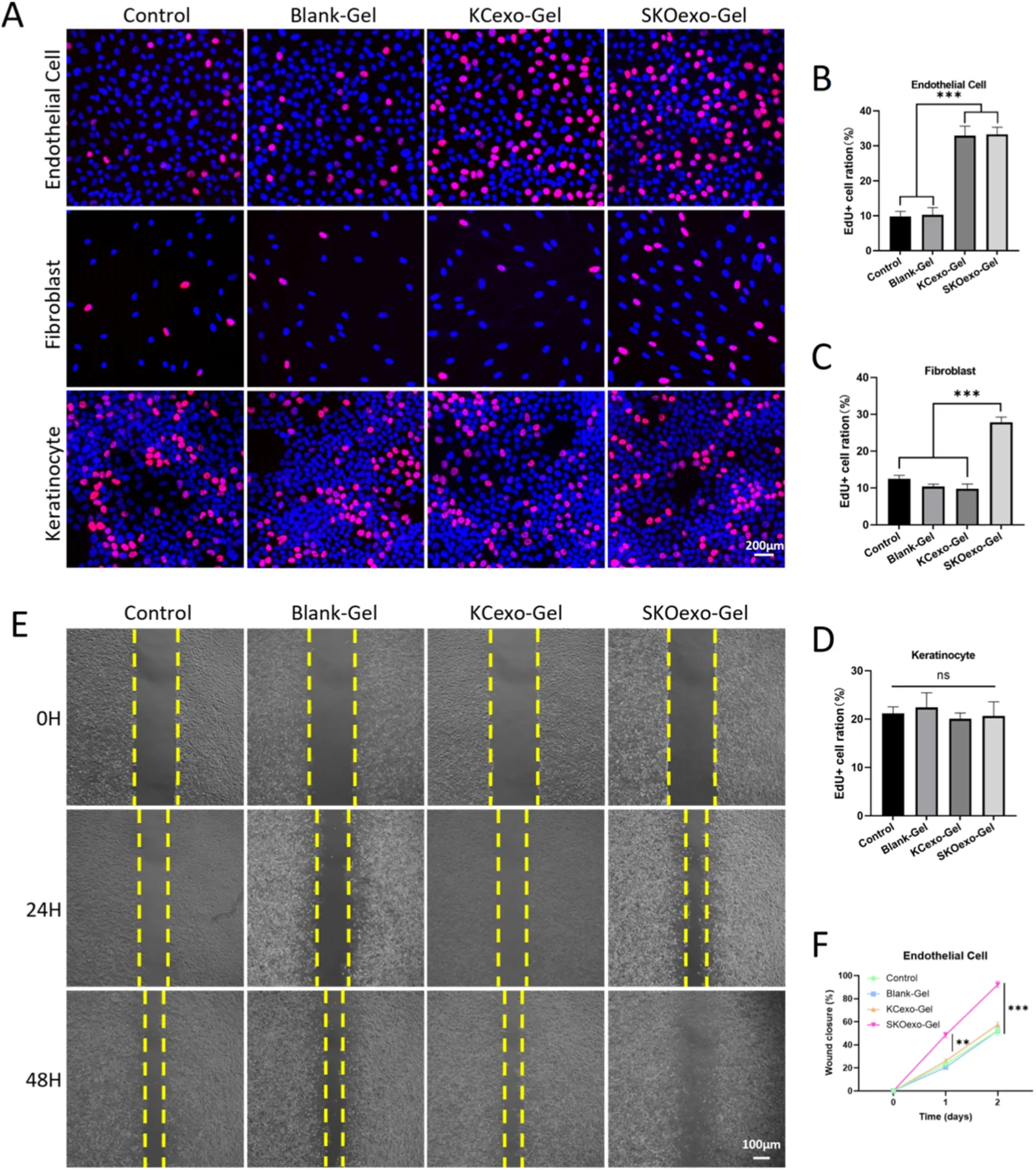
| **Functional effects of exosome-embedded hydrogels on the proliferation and migration of key skin cell types. A,** Representative fluorescence microscopy images of EdU-stained primary human endothelial cells, human dermal fibroblasts, and human keratinocytes after treatment with PBS (Control), Blank-Gel, KCexo-Gel, or SKOexo-Gel. Nuclei are stained with DAPI (blue), and proliferating cells (EdU-positive) are labeled in pink. Scale bar: 200 μm. **B, C, D,** Quantitative analysis of the percentage of EdU-positive cells for ECs (b), HDFs (c), and KCs (d) under the indicated treatments. **E,** Representative phase-contrast micrographs of a scratch wound healing assay performed on ECs at 0, 24, and 48 h post-treatment. The initial wound edges are marked with yellow dashed lines. Scale bar: 100 μm. **F,** Quantification of the relative wound closure area of ECs. Statistical significance was determined by one-way ANOVA; Data are represented as mean ± SD (n = 3–4/group). *P < 0.05, **P < 0.01, ***P < 0.001. (For interpretation of the references to color in this figure legend, the reader is referred to the Web version of this article.)

The proliferative effects of the hydrogel systems on human umbilical vein endothelial cells (HUVECs), human dermal fibroblasts (HDFs), and human keratinocytes (HaCaTs) were assessed via EdU incorporation assays. Quantitative analysis revealed distinct, cell type-specific responses. In HUVECs, both KCexo-Gel and SKOexo-Gel significantly increased the percentage of EdU-positive cells compared to the Control and Blank-Gel groups (Fig. 3A and B). In HDFs, a significant pro-proliferative effect was observed only in the SKOexo-Gel group compared to the Control and Blank-Gel (Fig. 3A–C). In contrast, none of the hydrogel treatments, regardless of exosome loading, significantly altered the proliferation rate of HaCaT keratinocytes compared to the Control (Fig. 3A–D). A scratch-wound assay was performed to evaluate the effect on cell migration. Representative phase-contrast images at 0, 24, and 48 h post-scratch show that the SKOexo-Gel group exhibited the most rapid wound closure compared to all other groups in HUVECs (Fig. 3E and F). The migration rate was quantified by measuring the relative wound area over time. In contrast, no significant differences in the wound closure rates were observed among any of the treatment groups for either HDFs or HaCaTs (Fig. S3).

**Fig. 4:**
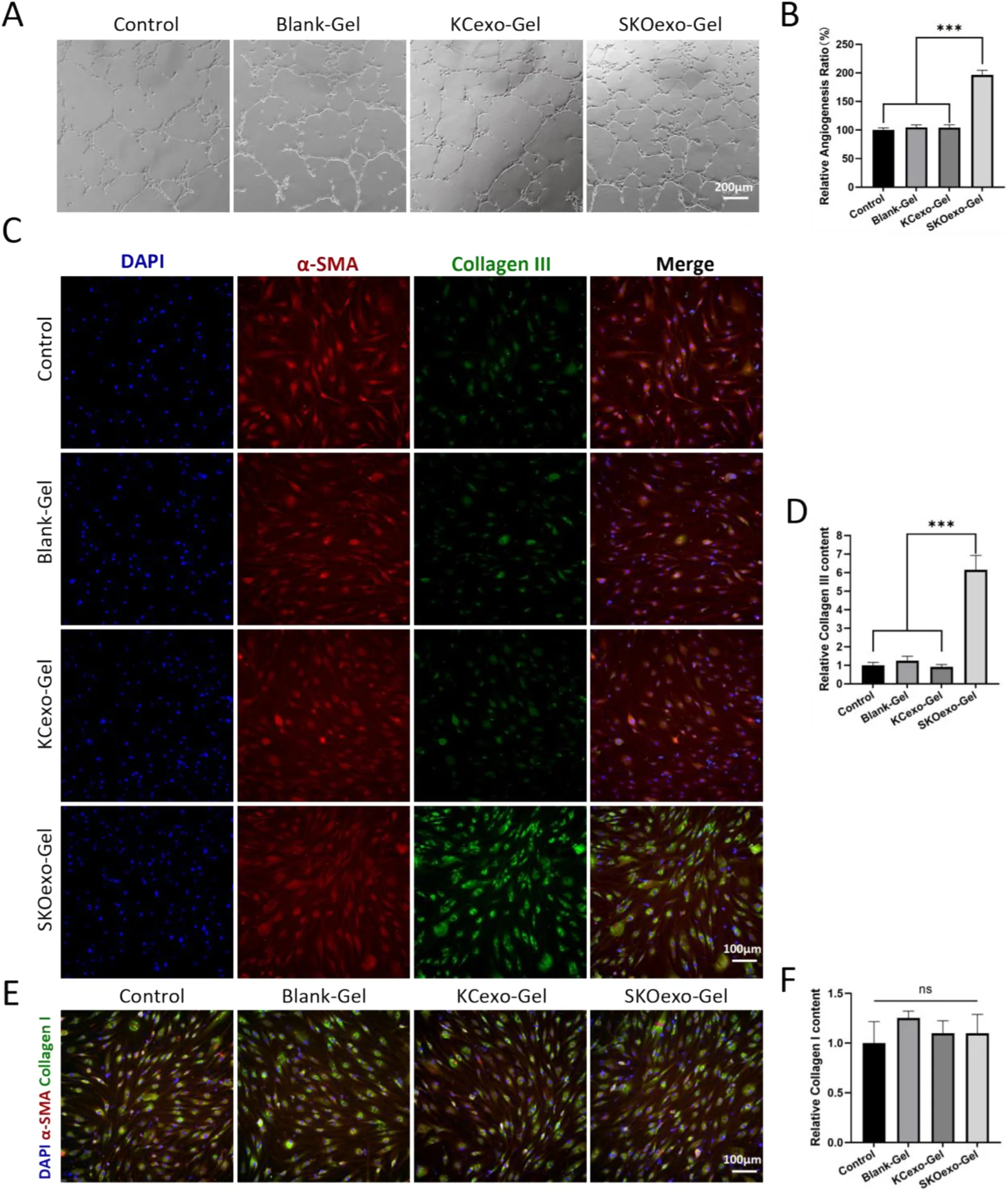
| **Functional effects of exosome-embedded hydrogels on the tubulogenesis and collagen secretion. A,** Representative images of in vitro tube formation assays using ECs treated with the conditioned medium from different hydrogel groups: Control (PBS), Blank-Gel, KCexo-Gel, and SKOexo-Gel. The formation of capillary-like tubular structures is shown. Scale bar: 200 μm. **B,** Quantitative analysis of the relative angiogenesis ratio, calculated from the total tube length in the assay. Data are presented as mean ± SD (n ≥ 3). **C, E,** Representative immunofluorescence images of cultured fibroblasts post-treatment. c, Staining for nuclei (DAPI, blue), α-SMA (red) and Collagen III (green). e, Staining for nuclei (DAPI, blue), α-SMA (red), and Collagen I (green). Scale bar: 100 μm. **D, F,** Quantitative analysis of the relative fluorescence intensity of (d) Collagen III and (f) Collagen I in the wound area. Statistical significance was determined by one-way ANOVA; Data are represented as mean ± SD (n = 3–4/group). *P < 0.05, **P < 0.01, ***P < 0.001. (For interpretation of the references to color in this figure legend, the reader is referred to the Web version of this article.)

The pro-angiogenic effect of the hydrogels systems was assessed using an endothelial cell tubulogenesis assay. Representative micrographs demonstrated that HUVECs cultured with SKOexo-Gel formed more extensive and well-connected tubular networks compared to the Control, Blank-Gel, and KCexo-Gel groups. Quantitative analysis of the relative angiogenesis ratio confirmed a significant increase in the SKOexo-Gel group, indicating its potent activity in promoting angiogenesis (Fig. 4A and B). The impact on collagen secretion by HDFs was evaluated via immunofluorescence staining. While alpha-smooth muscle actin (α-SMA) expression appeared consistent across groups, a marked increase in collagen III secretion was observed specifically in the SKOexo-Gel group. Quantitative analysis of the fluorescence intensity confirmed a significant upregulation of collagen III in the SKOexo-Gel group (Fig. 4C and D). In contrast, the secretion of collagen I was not significantly altered by any treatment, as shown by both representative images and quantitative data (Fig. 4E and F).

### 3.4 Evaluation of wound healing and hemostatic performance

**Fig. 5:**
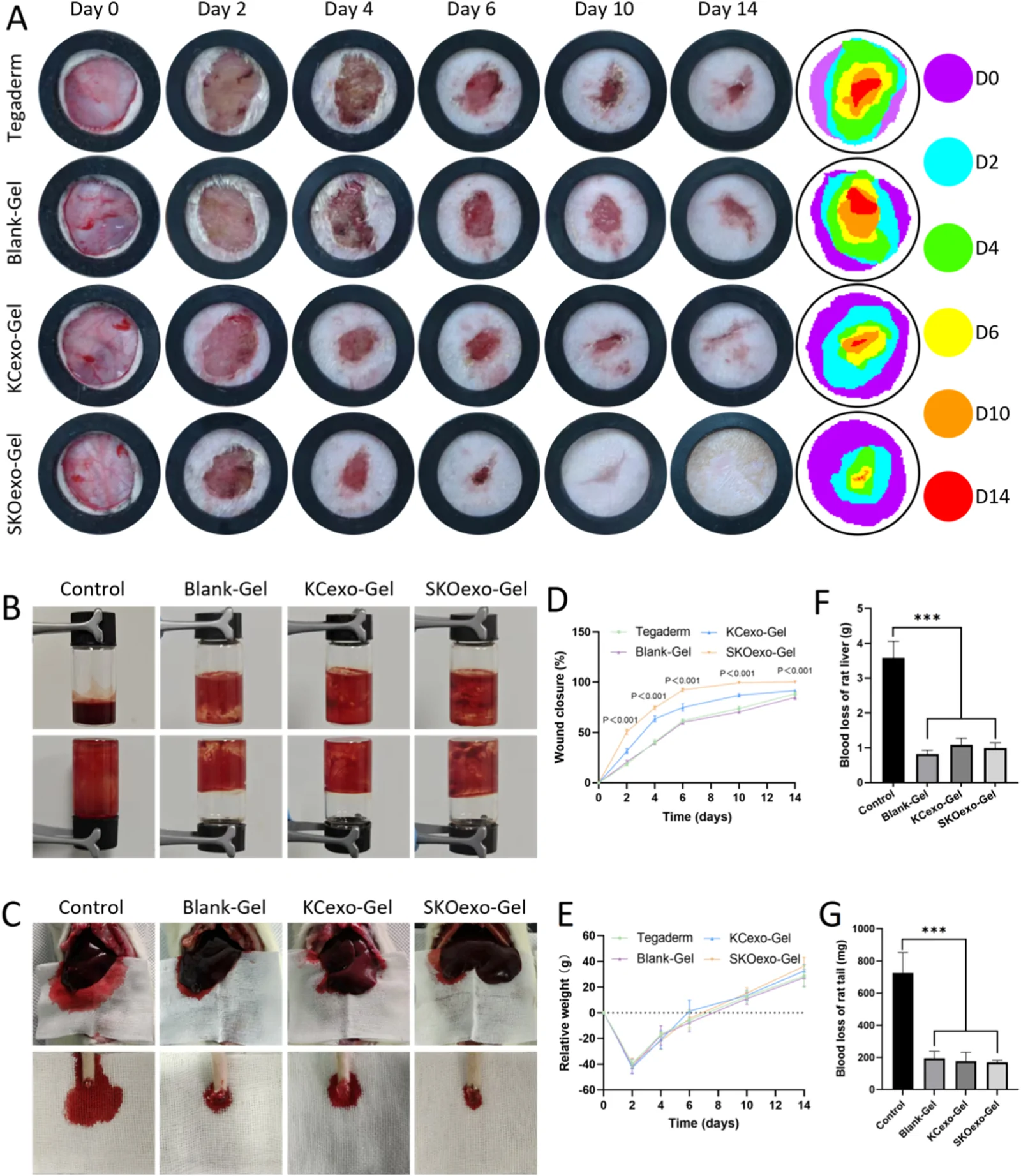
| **Characterization of acute wound healing and hemostatic capability in a rat model. A,** Photographs of excisional wounds taken on days 0, 2, 4, 6, 10, and 14 post-treatment, arranged to show the dynamic healing progression for rats treated with either Tegaderm film, Blank-Gel, KCexo-Gel, or SKOexo-Gel. The rightmost column illustrates the sequential reduction in wound area over time, with superimposed, color-coded outlines representing the wound edge at each corresponding time point (D0, purple; D2, cyan; D4, green; D6, yellow; D10, orange; D14, red), visually depicting the healing trajectory. **B,** In vitro procoagulant effects of the hydrogels assessed by a whole blood coagulation assay. **C,** In vivo hemostatic efficacy evaluated in a rat liver incision model and tail amputation model. **D,** Quantitative analysis of wound closure rates over the 14-day healing period. **E,** Line graph showing the changes in relative body weight of rat over a 14-day observation period. **F, G,** Statistical analyses of blood loss in the liver and tail models. Statistical significance was determined by one-way ANOVA; Data are represented as mean ± SD (n = 5–6/group). *P < 0.05, **P < 0.01, ***P < 0.001. (For interpretation of the references to color in this figure legend, the reader is referred to the Web version of this article.)

The wound healing efficacy of different treatments was first evaluated in an acute full-thickness excisional wound model over 14 days. Representative photographic images taken on days 0, 2, 4, 6, 10, and 14, along with color-coded area maps, are shown. Visually, wounds treated with SKOexo-Gel exhibited the most rapid reduction in wound size compared to the Tegaderm film, Blank-Gel, and KCexo-Gel groups. Quantitative analysis of the relative wound area over time confirmed that the SKOexo-Gel group achieved a significantly faster wound closure rate, reducing the wound area to approximately 50% on day 2, 70% on day 4, and nearly complete closure by day 10, outperforming all other groups. While the KCexo-Gel group demonstrated a modest pro-healing effect in the first ten days compared to the Tegaderm film and Blank-Gel groups, its efficacy was significantly lower than that of the SKOexo-Gel group, and no significant improvement was observed beyond day 10 compared to controls. These results indicate that SKOexo possesses a significant wound healing-promoting capacity both in the early and late stages of wound healing, and its efficacy is superior to that of KCexo. Meanwhile, the Blank-Gel group showed no discernible difference in wound closure dynamics compared to the Tegaderm film control, underscoring the critical role of the loaded exosomes, rather than the hydrogel matrix, in driving the observed pro-healing effects (Fig. 5A and D). Over the 14-day experimental period, all groups exhibited a comparable pattern of body weight change characterized by an initial decrease followed by a gradual recovery, with no statistically significant differences observed among the groups, indicating the absence of treatment-related systemic side effects (Fig. 5E). In the diabetic chronic wound model, SKOexo-Gel maintained a significantly enhanced wound closure rate throughout the entire 28-day healing cycle. In contrast, KCexo-Gel only exhibited accelerated healing within the first 3 days before transitioning into a stagnant, slow-healing trajectory similar to that of the other groups (Fig. S4).

The pro-coagulant activity of the hydrogel systems was evaluated using a whole blood coagulation assay in vitro. All hydrogel formulations—Blank-Gel, KCexo-Gel, and SKOexo-Gel—significantly accelerated clot formation compared to the Control group, demonstrating that the hydrogel matrix itself possesses intrinsic pro-coagulant properties. Critically, no notable differences in clotting kinetics or clot stability were observed among the three hydrogel groups, indicating that the incorporation of keratinocyte-derived or skin organoid-derived exosomes did not alter the inherent coagulation capability of the base hydrogel (Fig. 5B). The hemostatic efficacy was further validated in two distinct rat hemorrhage models. In a liver incision model, all hydrogel-treated groups exhibited a substantial and statistically comparable reduction in blood loss relative to the Control (Fig. 5C and F). Similarly, in a tail amputation model, all hydrogel groups achieved effective bleeding control, with no significant differences in blood loss observed among Blank-Gel, KCexo-Gel, and SKOexo-Gel (Fig. 5C and G). These consistent results across in vitro and in vivo assays demonstrate that the pro-coagulant activity is primarily attributable to the physicochemical properties of the hydrogel matrix—such as its porous network, which facilitates blood cell entrapment and platelet activation—rather than being influenced by the loaded exosomes.

### 3.5 Histological analysis of key repair processes in acute wound healing

**Fig. 6:**
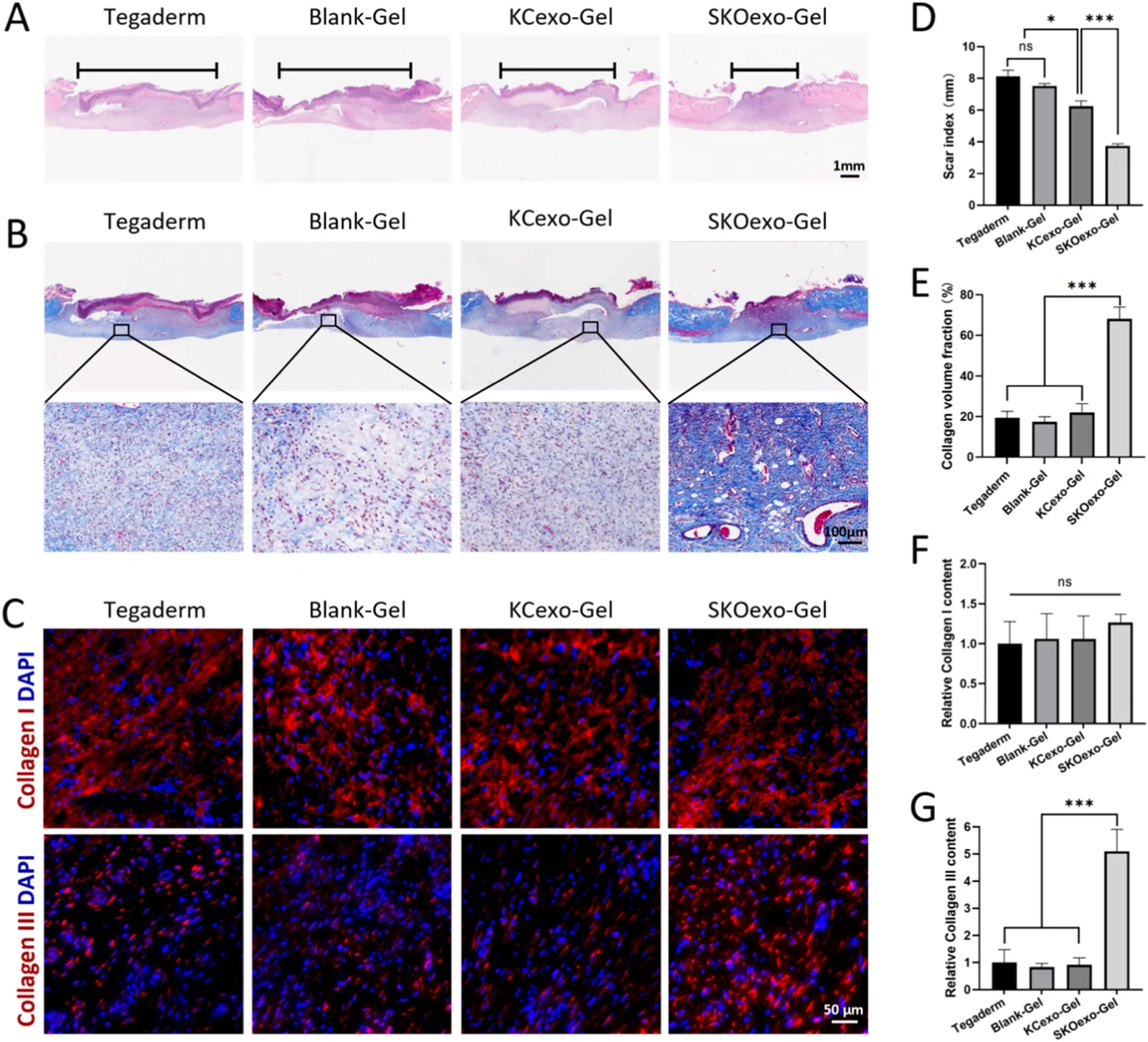
| **Histological assessment of wound healing and collagen deposition. A,** Representative H&E staining of full-thickness wound sections at day 7 post-treatment with Tegaderm film, Blank-Gel, KCexo-Gel, or SKOexo-Gel, showing overall tissue architecture and the extent of re-epithelialization. Scale bar: 1 mm. **B,** Representative Masson staining of full-thickness wound sections at day 7 post-treatment. Magnified views of the boxed areas providing detailed insight. Scale bar: 100 μm. **C,** Representative immunofluorescence staining of wound bed sections for extracellular matrix components: Collagen I (red, top row) and Collagen III (red, bottom row). Nuclei are counterstained with DAPI (blue). Scale bar: 50 μm. **D-G,** Quantitative analyses of histological parameters: (d) scar index, (e) collagen volume fraction, (f) relative Collagen I fluorescence intensity, and (g) relative Collagen III fluorescence intensity for the different treatment groups. Statistical significance was determined by one-way ANOVA; Data are represented as mean ± SD (n = 5–6/group). *P < 0.05, **P < 0.01, ***P < 0.001. (For interpretation of the references to color in this figure legend, the reader is referred to the Web version of this article.)

H&E staining of skin tissue sections revealed that acute wounds treated with SKOexo-Gel exhibited a smaller wound size compared to the Control, Blank-Gel, and KCexo-Gel groups at 7 days post-treatment. KCexo-Gel treatment resulted in a moderate improvement (Fig. 6A and D). Masson's trichrome staining confirmed significantly denser and more linearly organized collagen fibers in the SKOexo-Gel group, indicative of advanced matrix remodeling (Fig. 6B and E). To further delineate the composition of the newly deposited matrix, immunofluorescence staining for specific collagen types was performed. Analysis of Collagen I expression revealed no significant differences in fluorescence intensity among the Tegaderm, Blank-Gel, KCexo-Gel, and SKOexo-Gel groups (Fig. 6C and F). In contrast, the fluorescence intensity of Collagen III in the SKOexo-Gel group was significantly higher compared to all other treatment groups, which exhibited similarly low levels of Collagen III signal (Fig. 6C and G). These results indicate that the accelerated healing mediated by SKOexo-Gel is associated not merely with a general increase in collagen, but with a specific and pronounced promotion of Collagen III synthesis, a key collagen isoform involved in the early, regenerative phase of wound repair.

**Fig. 7:**
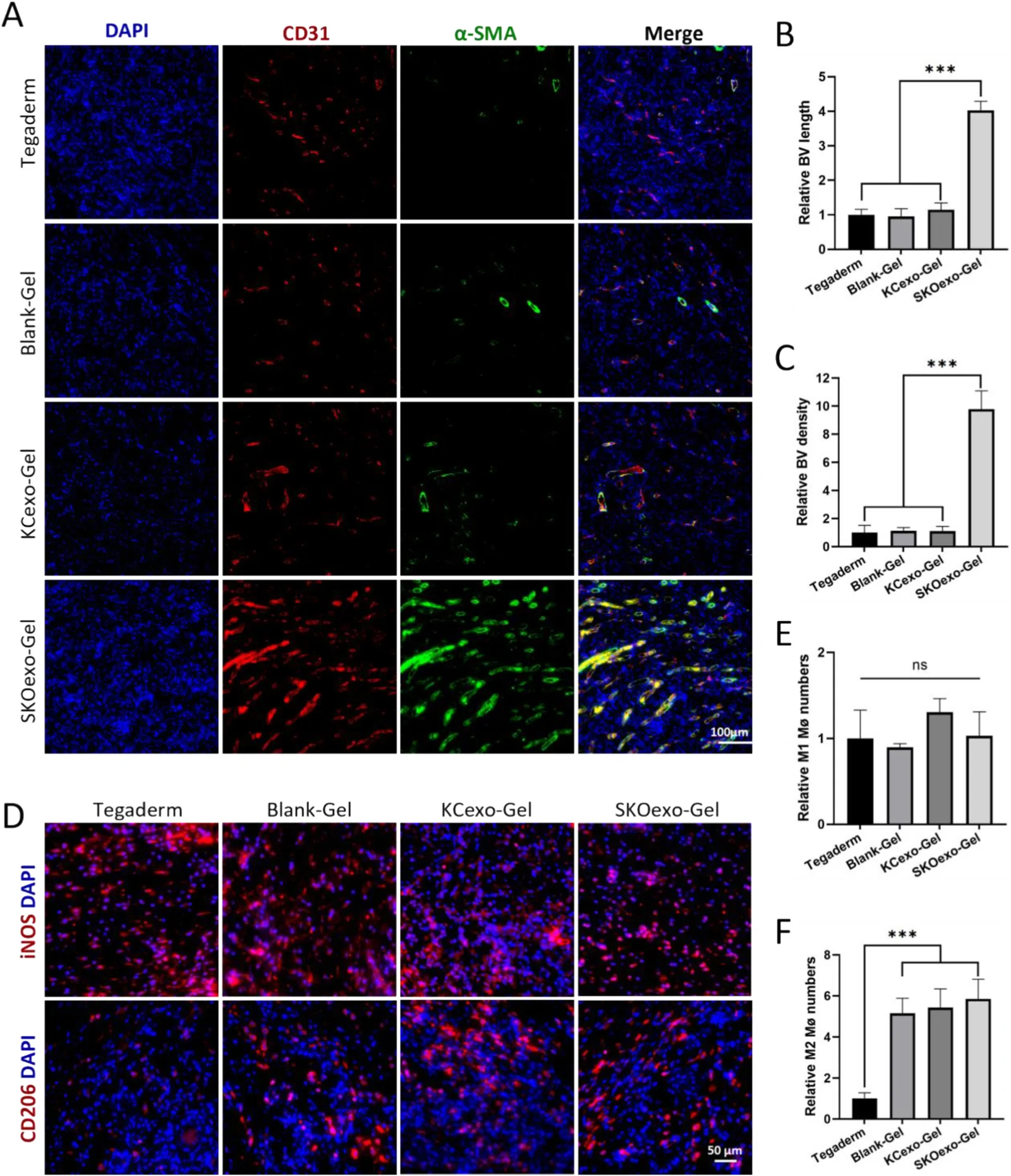
| **Histological assessment of angiogenesis and macrophage polarization in wound healing. A,** Representative immunofluorescence images of wound tissue sections at day 7, stained for the endothelial cell marker CD31 (red) and the myofibroblast/pericyte marker α-SMA (green). Nuclei are counterstained with DAPI (blue). Scale bar: 100 μm. **B, C,** Quantitative analysis of (b) relative blood vessel length and (c) vessel density based on CD31 staining. **D,** Representative immunofluorescence staining for the M1 macrophage marker iNOS (red, top row) and the M2 macrophage marker CD206 (red, bottom row) in wound sections. Nuclei are counterstained with DAPI (blue). Scale bar: 50 μm. **E, F,** Quantitative analysis of the relative numbers of (e) M1 (iNOS^+^) and (f) M2 (CD206^+^) macrophages. Statistical significance was determined by one-way ANOVA; Data are represented as mean ± SD (n = 5–6/group). *P < 0.05, **P < 0.01, ***P < 0.001. (For interpretation of the references to color in this figure legend, the reader is referred to the Web version of this article.)

Neovascularization is critical for successful wound repair, as it restores oxygen and nutrient supply to the healing tissue. Immunofluorescence staining for CD31 and α-SMA on wound tissues at day 7 was performed to evaluate the formation and maturity of newly formed blood vessels. Representative merged images demonstrated a marked increase in CD31^+^ structures within the granulation tissue of wounds treated with SKOexo-Gel compared to the Control, Blank-Gel, and KCexo-Gel groups. The newly formed vessels in the SKOexo-Gel group appeared more numerous, densely branched, and were frequently associated with α-SMA^+^ pericyte coverage, indicating enhanced vascular maturation (Fig. 7A–C). This robust pro-angiogenic effect observed in vivo aligns with and extends our previous in vitro findings, where SKOexo potently promoted endothelial cell proliferation, migration, and tube formation. Collectively, these results suggest that the potent wound-healing capacity of the SKOexo-Gel system is mediated, at least in part, through its strong ability to promote functional vascularization at the acute wound site.

A balanced immune response, particularly the timely transition of macrophages from a pro-inflammatory (M1) to a pro-healing (M2) phenotype, is crucial for effective wound repair. To assess the immunomodulatory effect of the treatments, immunofluorescence staining for iNOS^+^ (M1) and CD206^+^ (M2) macrophages was performed on acute wound tissues at day 7. Quantitative analysis revealed that none of the hydrogel-based treatments significantly altered the number of iNOS^+^ M1 macrophages compared to the Tegaderm control (Fig. 7D and E). Critically, while all hydrogel groups (Blank-Gel, KCexo-Gel, and SKOexo-Gel) showed a marked increase in CD206^+^ M2 macrophage infiltration compared to the Tegaderm control, no statistically significant difference in M2 macrophage numbers was observed among the three hydrogel formulations themselves (Fig. 7D and F). This result indicates that the hydrogel matrix provides a baseline immunomodulatory effect conducive to the M2 phenotype, but the incorporation of exosomes (from either keratinocytes or skin organoids) did not further quantitatively enhance this particular macrophage response at the time point assessed.

### 3.6 Identification of miRNA in SKO-exo via miRNA-seq and functional validation

**Fig. 8:**
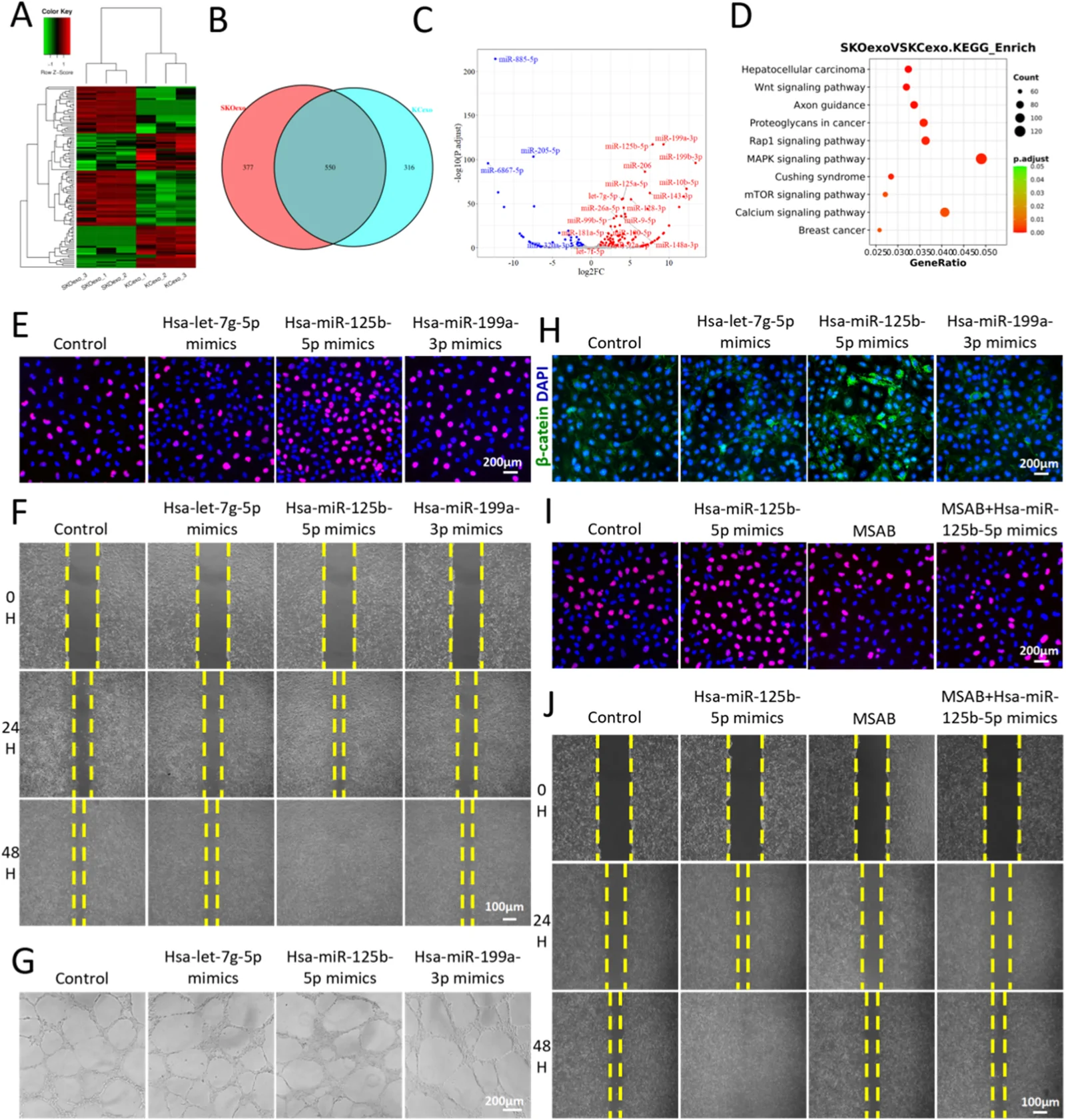
| **Bioinformatic and in vitro functional validation of key miRNAs enriched in SKOexo. A,** Heatmap of differentially expressed miRNAs in SKOexo compared to KCexo, showing a distinct enrichment profile. **B,** Venn diagram of miRNAs in SKOexo and KCexo. **C,** Volcano plot depicting significantly up- and down-regulated miRNAs (p-value < 0.05) in SKOexo. **D,** KEGG pathway enrichment analysis of predicted target genes for the enriched miRNAs, revealing their association with critical pathways. **E,** Representative fluorescence microscopy images of EdU assay in ECs treated with mimics for the top candidate miRNAs: Hsa-let-7g-5p, Hsa-miR-125b-5p, and Hsa-miR-199a-3p. Proliferating cells (EdU-positive) are shown in magenta. ‘Control’ indicates cells treated with scramble miRNA mimic. Scale bar: 200 μm. **F,** Representative phase-contrast images of scratch wound assays in ECs treated with the same miRNA mimics. Yellow dashed lines mark the initial wound edges. Scale bar: 100 μm. **G,** Representative phase-contrast images of tube formation assays in ECs treated with the indicated miRNA mimics. Scale bar: 200 μm. **H,** Immunofluorescence staining for β-catenin in EC treated with mimics for the candidate miRNAs Hsa-let-7g-5p, Hsa-miR-125b-5p, and Hsa-miR-199a-3p. β-catenin is shown in green and nuclei (DAPI) in blue. Scale bars: 200 μm. **I,** Proliferation assay in EC. Representative images of EdU staining (pink) for proliferating cells in EC treated as follows: scramble miRNA mimic (Control), Hsa-miR-125b-5p mimic, the Wnt/β-catenin pathway inhibitor MSAB alone, or a combination of Hsa-miR-125b-5p mimic and MSAB. Nuclei are counterstained with DAPI (blue). **J,** Migration assay in EC. Representative phase-contrast images of a scratch wound assay performed on EC treated as indicated in panel I. The initial wound edges are marked with yellow dashed lines. Scale bars for panels I and J are shown on the images. (For interpretation of the references to color in this figure legend, the reader is referred to the Web version of this article.)

To elucidate the molecular basis for the superior wound-healing efficacy of SKO-exo compared to KC-exo, small RNA sequencing was performed. Unsupervised hierarchical clustering of all detected miRNAs based on their normalized expression levels demonstrated a clear and complete separation between the SKO-exo and KC-exo groups, indicating fundamentally distinct miRNA cargo compositions (Fig. 8A). A Venn diagram analysis quantified this distinction, revealing that while a substantial core set of miRNAs was commonly packaged into both exosome types, SKO-exo contained a significantly larger population of exclusively detected miRNAs compared to KC-exo (Fig. 8B). Further analysis of the miRNA distribution revealed a markedly skewed profile in KC-exo, where a single miRNA, hsa-miR-885-5p, constituted over 50% of the total aligned reads, indicating a relatively limited miRNA repertoire. In contrast, SKO-exo exhibited a more balanced and poly-diverse miRNA population, suggesting a broader spectrum of regulatory molecules and a more complex functional capacity, potentially reflective of its multicellular tissue origin. The expression of differentially expressed miRNAs was visualized using a volcano plot (Fig. 8C). To infer the potential biological impact of this skewed miRNA profile, we performed KEGG pathway enrichment analysis on the predicted target genes of the SKO-exo-upregulated miRNAs. The analysis revealed a significant co-enrichment of disease-associated pathways and fundamental signaling pathways, indicating that these miRNAs predominantly target a core network of signaling circuits that share fundamental regulatory circuits with tissue repair processes. Notably, the Wnt signaling pathway emerged as the top enriched pathway. The significant enrichment of the Wnt pathway, a master regulator of development and regeneration, provides a compelling mechanistic hypothesis for the observed pro-angiogenic and pro-healing phenotypes, as Wnt signaling is intimately involved in endothelial cell behavior and skin repair (Fig. 8D).

Based on the consistent pro-angiogenic effects of SKO-exo observed in vitro and in vivo, we sought to identify the specific miRNA mediators responsible for this activity. Differential expression analysis identified a set of miRNAs significantly upregulated in SKO-Exo (FDR < 0.05). Among these, the top 10 most abundant miRNAs in SKO-Exo were selected for qPCR validation using independent exosome preparations. By applying a stringent threshold of ≥ 100-fold higher expression in SKO-Exo relative to KC-Exo, we identified three candidate miRNAs: hsa-miR-125b-5p, hsa-let-7g-5p, and hsa-miR-199a-3p (Table S1). Bioinformatic analysis indicating that the predicted target genes of these upregulated miRNAs are significantly enriched in pathways critically involved in vascular development and remodeling, suggesting a systematic approach to pinpointing key functional miRNAs. The selected miRNA mimics were then tested for their ability to modulate key endothelial cell functions. An EdU incorporation assay revealed that transfection with the Hsa-miR-125b-5p mimic significantly increased the proportion of proliferating HUVECs. In contrast, mimics for Hsa-let-7g-5p and Hsa-miR-199a-3p did not promote significant cell proliferation (Fig. 8E–S5A). This pro-proliferative effect was consistent with a pro-migratory function: a scratch-wound assay demonstrated that the Hsa-miR-125b-5p mimic also significantly enhanced HUVEC migration, while the other two mimics showed no significant effect (Fig. 8F–S5B). However, in an endothelial tube formation assay, none of the three miRNA mimics, including Hsa-miR-125b-5p, induced a significant increase in capillary-like network formation compared to the control (Fig. 8G–S5C). Collectively, these results identify Hsa-miR-125b-5p as a notable SKO-exo-enriched miRNA capable of promoting endothelial cell proliferation and migration in vitro, partially recapitulating the pro-angiogenic activity of the parent exosomes.

Building on the functional identification of Hsa-miR-125b-5p, we next investigated its underlying mechanism. Given the prominent enrichment of the Wnt/β-catenin signaling pathway among the predicted targets of SKO-exo miRNAs (Fig. 8D), we hypothesized that miR-125b-5p might exert its effects by modulating this pathway. Immunofluorescence analysis for β-catenin demonstrated that transfection with the miR-125b-5p mimic, but not with mimics for Hsa-let-7g-5p or Hsa-miR-199a-3p, led to a pronounced and widespread upregulation of β-catenin protein levels throughout HUVECs, indicating a robust activation of the Wnt/β-catenin signaling pathway (Fig. 8H–S5D). To functionally link this pathway activation to the observed cellular phenotypes, we employed MSAB, a specific inhibitor of β-catenin-mediated transcription. Consistent with the pro-migratory effect, the EdU incorporation assay confirmed that the pro-proliferative activity of miR-125b-5p was also dependent on β-catenin signaling, as co-treatment with MSAB completely abolished the increase in EdU-positive cells induced by the miR-125b-5p mimic (Fig. 8I–S5E). Similarly, the pro-migratory effect of miR-125b-5p, as assessed by a scratch-wound assay, was completely abrogated in the presence of MSAB (Fig. 8J–S5F). These functional rescue experiments demonstrate that the ability of miR-125b-5p to promote both endothelial cell proliferation and migration is dependent on active β-catenin signaling. These data mechanistically connect the SKO-exo-enriched miR-125b-5p to the activation of the Wnt/β-catenin pathway and establish this axis as a critical mediator of its pro-angiogenic activity.

To further investigate the functional redundancy and specificity within the top 10 abundant miRNAs, we extended our functional assays to the remaining seven candidates. Notably, while our preceding studies demonstrated that hsa-miR-125b-5p exhibited partial activity in promoting proliferation and migration but not tube formation, our investigation of the remaining seven miRNAs revealed that hsa-miR-99b-5p mimics significantly enhanced tube formation, and a few individual miRNAs also facilitated EC proliferation or migration. Collectively, these findings suggest that the pro-healing effects of SKO-Exo are likely not driven by a single miRNA, but rather by the composite and synergistic actions of the entire exosomal miRNA cargo, constituting a systemic pro-healing phenotype (Fig. S6).

## 4 Discussion

The management of skin wounds, whether acute or chronic, remains a significant clinical challenge, necessitating the development of innovative therapeutic strategies that can effectively orchestrate the complex multi-phase process of wound healing [23]. Cell-free therapies, particularly exosomes derived from stem cells, have emerged as a promising alternative to whole-cell therapies, offering advantages such as reduced immunogenicity, enhanced stability, and avoidance of ethical concerns [24,25]. These nano-sized vesicles serve as natural carriers of bioactive molecules, including proteins, lipids, and nucleic acids, capable of modulating key wound healing processes such as angiogenesis, epithelialization, and immune regulation [[26], [27], [28]]. While mesenchymal stem cell (MSC)-derived exosomes have been widely investigated for wound healing, their therapeutic efficacy may be limited by their inability to fully replicate the complex, multi-cellular microenvironment of native skin [29]. In this context, skin organoids represent a groundbreaking advance, as they recapitulate the hierarchical structure and cellular heterogeneity of native skin, including keratinocytes, fibroblasts, and developing appendages [20]. We hypothesized that exosomes derived from these skin organoids would be enriched with a tissue-specific cargo that more effectively coordinates the interdependent phases of wound repair compared to exosomes from homogeneous cell cultures, such as 3D keratinocytes. Our results strongly support this hypothesis, demonstrating that SKO-Exo loaded into a hydrogel (SKOexo-Gel) significantly outperformed KC-Exo loaded hydrogel (KCexo-Gel) in accelerating wound closure, promoting functional angiogenesis, and enhancing collagen III deposition in a full-thickness acute wound model.

The superior efficacy of SKOexo-Gel is likely attributable to the distinct biomolecular cargo of SKO-exo, which originates from a more physiologically relevant, multi-cellular source. Our in vitro functional assays revealed that SKOexo-Gel, but not KCexo-Gel, significantly promoted the proliferation of human dermal fibroblasts, and both exosome-loaded hydrogels enhanced human umbilical vein endothelial cell proliferation. Crucially, only SKOexo-Gel potently stimulated HUVEC migration and the formation of extensive, well-connected tubular networks, indicating a specific pro-angiogenic capacity that KC-exo lacked. This aligned with our in vivo findings, where acute wounds treated with SKOexo-Gel exhibited a significantly higher density of CD31^+^ blood vessels with enhanced pericyte coverage (α-SMA^+^), indicative of mature and functional vasculature [[30], [31], [32]]. Furthermore, the selective upregulation of collagen III, a key component of early, regenerative matrix deposition [33,34], specifically in the SKOexo-Gel group, underscores the ability of SKO-exo to promote a more organized and pro-regenerative healing response compared to the more limited effects of KC-exo. This precise, cell-type-specific regulation of dermal remodeling addresses a critical limitation of conventional single-cell derived exosomes. Unlike MSC-Exo, which broadly drives keratinocyte hyperproliferation and often triggers excessive type I collagen deposition (a pathological hallmark of fibrosi), SKO-Exo selectively amplifies fibroblast activity and collagen III secretion without disrupting the homeostatic balance of collagen I or epidermal proliferation. This biomimetic phenotype mirrors the physiological wound healing cascade, wherein a transient, flexible collagen III matrix matures into organized tissue rather than disorganized scars.

The enhanced functional capacity of SKO-exo is undoubtedly linked to its more complex and diverse molecular composition. Our miRNA sequencing analysis provided compelling evidence for this, revealing a fundamental disparity between the miRNA profiles of SKO-exo and KC-exo. While KC-Exo exhibited a remarkably skewed miRNA profile dominated by a single species (hsa-miR-885-5p), SKO-Exo presented a balanced and poly-diverse population of miRNAs. This suggests that the therapeutic effect of SKO-exo is not driven by a single, highly abundant molecule, but rather by the synergistic action of a broad spectrum of regulatory molecules, more accurately mirroring the complex cell-to-cell communication occurring in native skin tissue. This diversity likely enables SKO-exo to simultaneously target multiple signaling pathways involved in proliferation, migration, matrix synthesis, and immune modulation, thereby achieving a more coordinated and robust healing outcome.

Adequate neovascularization and restoration of local perfusion are widely recognized as a core determinant of successful cutaneous wound repair, as robust blood supply sustains granulation tissue maturation and re-epithelialization—a priority explicitly underscored in recent benchmark exosome-hydrogel studies for wound repair [35]. Driven by the prominent pro-angiogenic phenotype of SKOexo-Gel, we sought to identify specific miRNA mediators within SKO-exo. Our selection of three miRNAs, Hsa-let-7g-5p, Hsa-miR-125b-5p, and Hsa-miR-199a-3p, was based on their significant enrichment in SKO-Exo compared to KC-Exo. Hsa-let-7g-5p, a member of the let-7 family, is known to influence cell cycle progression and has been implicated in promoting endothelial cell function and angiogenesis under certain conditions [[36], [37], [38]]. Hsa-miR-199a-3p has been reported to exhibit complex, context-dependent roles in angiogenesis, often modulating hypoxia-inducible factor (HIF) pathways and vascular endothelial growth factor (VEGF) signaling [39,40]. Most compellingly, Hsa-miR-125b-5p was prioritized due to its established reputation as a key regulator of vascular biology, with prior studies showing it can enhance endothelial cell proliferation and migration by targeting negative regulators of growth factor signaling [[41], [42], [43]]. Our focused investigation identified Hsa-miR-125b-5p as a prominent functional contributor. We demonstrated that miR-125b-5p mimic transfection significantly promoted both the proliferation and migration of HUVECs, partially recapitulating the pro-angiogenic activity of the parent exosomes. This finding aligns with and extends the existing literature on miR-125b-5p′s role in vascular repair. Mechanistically, we linked this effect to the activation of the Wnt/β-catenin signaling pathway, a master regulator of development and regeneration [[44], [45], [46]]. The enrichment of Wnt signaling pathway among the predicted targets of SKO-exo miRNAs, coupled with our functional validation showing that the pro-proliferative and pro-migratory effects of miR-125b-5p were entirely dependent on β-catenin-mediated transcription, provides a clear mechanistic axis for at least part of SKO-Exo's therapeutic action. While the individual roles of let-7g-5p and miR-199a-3p in our specific assays were less pronounced, their presence in the complex cargo of SKO-Exo suggests potential synergistic or context-dependent contributions to the overall healing response, a notion supported by their known involvement in other aspects of tissue repair. Notably, the enrichment of miR-125b-5p and its subsequent activation of the Wnt/β-catenin axis distinguishes the SKO-Exo signature from that of conventional MSC-Exo. While MSC-Exo typically prioritizes VEGF or TGF-β signaling to achieve general tissue repair, SKO-Exo leverages this developmental Wnt signaling program to coordinate multiple regenerative processes simultaneously. This broader regulatory scope allows SKO-Exo to integrate vascular reconstruction with the precise dermal remodeling described earlier, moving beyond the single-target approach that often limits current therapies. Thus, our findings not only validate a specific miRNA-pathway axis but also highlight the advantage of SKO-Exo's multicellular origin, which packages a consortium of miRNAs capable of orchestrating a coordinated regenerative program.

While Hsa-miR-125b-5p was identified as a contributor to angiogenesis, our tube formation assay indicated that it alone was insufficient to fully replicate the complex angiogenic program induced by the whole exosomes. Hsa-miR-99b-5p can significantly promoted tube formation. This strongly implies that the comprehensive wound-healing effects of SKO-Exo are the product of a synergistic combination of multiple miRNAs, other nucleic acids, proteins, and lipids acting in concert. The inability of a single miRNA mimic to fully phenocopy the exosome's effect underscores the concept that organoid-derived exosomes function as a unified biological delivery system, whose therapeutic power lies in the complexity of their cargo. Future studies employing gain-of-function and loss-of-function screens for a broader set of SKO-Exo-enriched miRNAs, as well as proteomic and lipidomic analyses, will be essential to deconvolute this sophisticated network of interacting molecules.

Beyond the exosomal cargo, the hydrogel delivery system itself played a critical role in the therapeutic success. The PVA/gelatin/boric acid hydrogel provided a biocompatible, adhesive, and self-healing scaffold that ensured the sustained release of exosomes with an initial burst, thereby maintaining a therapeutically relevant concentration at the wound site. Importantly, the hydrogel matrix itself contributed to a pro-healing microenvironment, as evidenced by the increased infiltration of M2 macrophages in all hydrogel-treated groups compared to the Tegaderm control [[47], [48], [49]]. This suggests that the hydrogel creates a favorable niche for tissue repair, upon which the specific biological instructions carried by the exosomes (particularly SKO-Exo) act to dramatically enhance the quality and speed of healing. The hemostatic properties of the hydrogel, independent of exosome loading, further highlight its utility for acute wound management. Beyond serving as a passive delivery vehicle, the PVA/gelatin/boric acid hydrogel likely engages in multifaceted interactions with the loaded exosomes that contribute to the observed therapeutic efficacy. The hydrogel's porous microstructure provides a protective microenvironment that shields exosomes from enzymatic degradation and mechanical shear at the wound site. Furthermore, the self-healing property of the hydrogel allows dynamic reorganization of the polymer network in response to wound contraction, which may actively promote exosome release into the regenerating tissue rather than relying solely on passive diffusion. These synergistic interactions—physical protection, sustained presentation, and responsive release—collectively enable the hydrogel to function not merely as a carrier but as a bioactive scaffold​ that potentiates the therapeutic effects of SKO-Exo.

It is worth noting that our hydrogel system does not possess intrinsic antibacterial activity, as confirmed by in vitro antibacterial assays. This finding highlights the necessity of regular hydrogel replacement during in vivo treatment to prevent bacterial colonization and ensure therapeutic efficacy. Indeed, periodic dressing changes are a standard practice in clinical wound management, particularly for chronic wounds. Looking ahead, we aim to develop advanced hydrogel delivery systems with potent antibacterial properties to further benefit patients, especially those suffering from chronic and refractory wounds.

Of particular interest is our observation that the SKOexo-Gel treatment significantly promoted the proliferation of HDFs but did not exert a notable pro-proliferative effect on HaCaTs in vitro. A critical consideration is that while SKO-Exo promoted HDF proliferation, the pathogenesis of fibrotic scarring (such as in hypertrophic scars and keloids) involves not merely an increase in fibroblast number but, more importantly, a functional dysregulation characterized by excessive production and impaired degradation of a Type-I-collagen-dominated extracellular matrix (ECM) [[50], [51], [52]]. Our findings reveal that SKO-Exo, while stimulating HDF proliferation, concomitantly and specifically upregulated their secretion of Collagen III without a significant effect on Collagen I, both in vitro and in vivo. This directed modulation of the secretory profile towards a Collagen-III-rich matrix suggests a mechanism that may help strike a balance between rapidly filling the wound defect (requiring cell proliferation) and promoting normal matrix remodeling with a more physiological collagen ratio, thereby favoring regenerative healing over fibrotic scarring. Collagen III is a major component of the embryonic ECM and early granulation tissue, forming a finer, more elastic network, whereas a high Collagen I/III ratio is a hallmark of scar tissue [53,54]. The SKO-Exo-induced phenotype, stimulating fibroblast activity while specifically guiding them towards a Collagen-III-productive state, likely contributes to the observed high-quality healing by promoting the restoration of a more physiological dermal architecture. This nuanced perspective is further highlighted when contrasting our findings with the broader literature on exosome therapy, where numerous studies have reported that exosomes derived from MSCs consistently promote the proliferation and migration of keratinocytes, accelerating re-epithelialization as a primary healing mechanism [[55], [56], [57]]. The absence of a significant mitogenic effect on HaCaTs by SKO-Exo, coupled with its unique modulation of fibroblast function, underscores a crucial source-dependent specificity. The cargo of SKO-Exo, originating from a multicellular skin organoid that mimics native tissue heterogeneity, likely delivers a more complex set of instructions that coordinately prioritize robust dermal restoration and angiogenesis rather than indiscriminately driving the proliferation of all cutaneous cell types. This targeted action might be advantageous in orchestrating a balanced healing process. Future investigations utilizing chronic wound models or assays probing long-term collagen maturation and keratinocyte differentiation will be essential to fully elucidate the role of SKO-Exo in preventing aberrant scarring and orchestrating epidermal regeneration. The distinct biological activity of organoid-derived exosomes underscores their potential as a next-generation therapy capable of fine-tuning the wound healing process.

## 5 Conclusion

Our study introduces a novel and highly effective strategy for wound healing by combining the biomimetic advantages of skin organoid-derived exosomes with the sustained-release capability of a hydrogel. We provide compelling evidence that SKO-Exo possess superior wound-healing capabilities compared to exosomes from a simpler cellular source, attributable to their more complex and tissue-specific cargo. We have delineated a specific mechanism involving the SKO-Exo-enriched miR-125b-5p and its activation of the Wnt/β-catenin pathway in promoting angiogenesis. While recognizing the limitation of focusing on a subset of miRNAs, our work underscores the paradigm that organoid-derived exosomes, as multifaceted biological entities, hold great promise as next-generation therapeutics. Future research exploring the full spectrum of their molecular components and their synergistic actions will be vital for unlocking their complete therapeutic potential and advancing their clinical translation for the treatment of wounds.

## CRediT authorship contribution statement

**Tao Yang:** Writing – original draft, Validation, Software, Methodology, Investigation, Formal analysis, Data curation, Conceptualization. **Wen Zheng:** Supervision, Methodology, Investigation, Formal analysis, Data curation. **Yue Li:** Resources, Methodology. **Yingshi Li:** Software, Methodology, Data curation. **Zhuxian Wang:** Validation, Supervision, Methodology. **Qiuyu Chen:** Formal analysis, Data curation. **Yangmeihui Wang:** Methodology. **Xiaoye Shi:** Methodology. **Jun Liu:** Writing – review & editing, Validation, Supervision, Investigation, Formal analysis, Conceptualization. **Bin Yang:** Validation, Supervision, Resources, Methodology, Investigation, Funding acquisition, Conceptualization.

## Funding

This research was funded by the 10.13039/501100001809National Natural Science Foundation of China (Grant No. 82473506).

## Declaration of competing interest

The authors declare that they have no known competing financial interests or personal relationships that could have appeared to influence the work reported in this paper.

## Data Availability

Data will be made available on request.
