# Supplementary material for: Skin organoid-derived exosomes in a hydrogel system potentiate skin wound healing
Source: Mater Today Bio. 2026 Sep 1;40:103632. doi: 10.1016/j.mtbio.2026.103632 (PMC13571936; doi:10.1016/j.mtbio.2026.103632)
Supplement: Supplementary file 1 [file mmc1.docx]

**Fig. S1 | Western blot analysis of GM130, Calnexin, and GAPDH in cells and exosomes derived from KC and SKO.**

**Fig. S2 | Antibacterial efficacy against common wound pathogens.**

**A,** Representative agar plates showing bacterial growth of S. aureus, P. aeruginosa, and MRSA after treatment with PBS, 4 μg/ml Ampicillin, Blank-Gel, KCexo-Gel, or SKOexo-Gel. **B,**  Quantified bacterial viability (% content) for each group. Statistical significance was determined by one-way ANOVA; Data are represented as mean±SD (n = 3-4/group). *P < 0.05, **P < 0.01, ***P < 0.001.

**Fig. S3 | Scratch wound assay on fibroblasts and keratinocytes treated with the hydrogel system.**

**A, C,** Representative phase-contrast micrographs of scratch wound healing assays performed on human dermal fibroblasts (A) and human keratinocytes (C) at 0, 24, and 48 hours post-treatment with PBS (Control), Blank-Gel, KCexo-Gel, or SKOexo-Gel. The initial wound edges are marked with yellow dashed lines. Scale bar: 100 μm. **B, D,** Quantitative analysis of the wound closure percentage for fibroblasts (B) and keratinocytes (D) . Statistical significance was determined by one-way ANOVA; Data are represented as mean±SD (n = 5–6/group). *P < 0.05, **P < 0.01, ***P < 0.001.

**Fig. S4 | In vivo evaluation of diabetic wound healing in a rat model.**

**A,** Schematic illustration of the animal experimental protocol for diabetic wound healing assessment. **B,** Representative wound images (Days 0, 3, 7, 14, 21, 28) and thermal images (right) of rats treated with Tegaderm, Blank-Gel, KCexo-Gel, or SKOexo-Gel, with color-coded wound outlines (D0–D28) showing healing progression. **C,** Quantitative wound closure rates over 28 days . **D,** Body weight changes throughout the entire experimental period. Statistical significance was determined by one-way ANOVA; Data are represented as mean±SD (n = 5–6/group). *P < 0.05, **P < 0.01, ***P < 0.001.

**Fig. S5 | Quantitative analysis of endothelial cell proliferation, migration, and associated signaling.**

**A,E** Quantitative analysis of EC proliferation from EdU assays, presented as the percentage of EdU+ cells. **B,** **F** Quantitative analysis of EC migration from scratch-wound assays, presented as the relative wound closure area. **C,** Quantitative analysis of in vitro tube formation, presented as the relative angiogenesis ratio. **D,** Quantitative analysis of β-catenin expression levels, determined by immunofluorescence intensity. In all panels, 'Control' refers to cells treated with a scramble miRNA mimic. Statistical significance was determined by one-way ANOVA; Data are represented as mean±SD (n = 5–6/group). *P < 0.05, **P < 0.01, ***P < 0.001.

**Fig. S6 | Evaluation of the remaining seven microRNAs on EC proliferation, migration, and tube formation.**

**A,** EdU incorporation assay showing cell proliferation rates. **B,** Wound healing assay at 0 h, 24 h, and 48 h post-scratch. **C,** Tube formation assay on Matrigel, showing capillary-like structures formed by endothelial cells. **D-F,** Quantification of the functional assays. Statistical significance was determined by one-way ANOVA; Data are represented as mean±SD (n = 4–5/group). *P < 0.05, **P < 0.01, ***P < 0.001.

**miRNA Screening and Validation Summary**

| **No.** | **miRNA** | **SKOexo_1**  **(Raw Read Counts)** | **qPCR Fold Change**  **(SKO/KC)** | **Selected** |
| --- | --- | --- | --- | --- |
| 1 | hsa-miR-125b-5p | 3643 | **260×** | **Yes** |
| 2 | hsa-let-7g-5p | 3369 | **165×** | **Yes** |
| 3 | hsa-miR-199a-3p | 2787 | **696×** | **Yes** |
| 4 | hsa-miR-204-5p | 2728 | 64× | No |
| 5 | hsa-miR-99b-5p | 2706 | 52× | No |
| 6 | hsa-miR-125a-5p | 2493 | 18× | No |
| 7 | hsa-miR-100-5p | 2004 | 6× | No |
| 8 | hsa-miR-206 | 1594 | 13× | No |
| 9 | hsa-let-7f-5p | 1574 | 6× | No |
| 10 | hsa-miR-26a-5p | 1529 | 9× | No |

**Table S1. Stepwise selection of candidate miRNAs for functional validation.** Top 10 abundant miRNAs in SKO-Exo were shortlisted from miRNA-seq and independently validated by qPCR . The qPCR-derived fold change (SKO-Exo / KC-Exo) was used as the final cutoff (≥100-fold), yielding three candidates (highlighted in green) for comprehensive functional assays.
